# Determinants of targeted cancer therapy use in community oncology practice: a qualitative study using the Theoretical Domains Framework and Rummler-Brache process mapping

**DOI:** 10.1186/s43058-023-00441-3

**Published:** 2023-06-12

**Authors:** Shellie D. Ellis, Joanna Veazey Brooks, Sarah A. Birken, Emily Morrow, Zachary S. Hilbig, Elizabeth Wulff-Burchfield, Anita Y. Kinney, Edward F. Ellerbeck

**Affiliations:** 1 University of Kansas School of Medicine, 3901 Rainbow Blvd., Kansas City, KS 66610 USA; 2 Wake Forest University School of Medicine, 525 Vine Street, Winston-Salem, NC 27101 USA; 3 Kansas City Kansas Community College, 7250 State Ave., Kansas City, KS 66112 USA; 4 Rutgers Cancer Institute of New Jersey, Rutgers University, 195 Little Albany St., New Brunswick, NJ 08901 USA

**Keywords:** Cancer care delivery, Precision medicine, Targeted cancer therapy, Genomic testing, Professional role and identity, Determinant analysis, Process mapping, Behavior specification, Community-based practice

## Abstract

**Background:**

Precision medicine holds enormous potential to improve outcomes for cancer patients, offering improved rates of cancer control and quality of life. Not all patients who could benefit from targeted cancer therapy receive it, and some who may not benefit do receive targeted therapy. We sought to comprehensively identify determinants of targeted therapy use among community oncology programs, where most cancer patients receive their care.

**Methods:**

Guided by the Theoretical Domains Framework, we conducted semi-structured interviews with 24 community cancer care providers and mapped targeted therapy delivery across 11 cancer care delivery teams using a Rummler-Brache diagram. Transcripts were coded to the framework using template analysis, and inductive coding was used to identify key behaviors. Coding was revised until a consensus was reached.

**Results:**

Intention to deliver precision medicine was high across all participants interviewed, who also reported untenable knowledge demands. We identified distinctly different teams, processes, and determinants for (1) genomic test ordering and (2) delivery of targeted therapies. A key determinant of molecular testing was role alignment. The dominant expectation for oncologists to order and interpret genomic tests is at odds with their role as treatment decision-makers’ and pathologists’ typical role to stage tumors. Programs in which pathologists considered genomic test ordering as part of their staging responsibilities reported high and timely testing rates. Determinants of treatment delivery were contingent on resources and ability to offset delivery costs, which low- volume programs could not do. Rural programs faced additional treatment delivery challenges.

**Conclusions:**

We identified novel determinants of targeted therapy delivery that potentially could be addressed through role re-alignment. Standardized, pathology-initiated genomic testing may prove fruitful in ensuring patients eligible for targeted therapy are identified, even if the care they need cannot be delivered at small and rural sites which may have distinct challenges in treatment delivery. Incorporating behavior specification and Rummler-Brache process mapping with determinant analysis may extend its usefulness beyond the identification of the need for contextual adaptation.

**Supplementary Information:**

The online version contains supplementary material available at 10.1186/s43058-023-00441-3.

Contributions to the literature

- The first study to identify the determinants of the delivery of precision medicine in community oncology settings, where most US cancer patients receive care
- Identifies role misalignment as a previously undiscovered, distinct barrier to genomic testing, creating justification for new implementation strategies to support targeted cancer therapy delivery by focusing on changes in professional roles among teams typically responsible for cancer care delivery, rather than knowledge needs of individual providers
- Expands the value of existing implementation science determinant frameworks, which emphasize the contextual adaptation needs, by explicit specification of the intervention behavior through process mapping methods
- Demonstrates the value of behavior specification in determinant analysis in addition to implementation strategy evaluation

## Background

Precision medicine is the practice of tailoring treatments to individual patients by classifying individuals into subpopulations that differ in their susceptibility to disease or response to treatment [1]. The promise of precision medicine lies in its ability to guide healthcare decisions toward the most effective treatment for a given patient, while reducing the need for unnecessary therapies, side effects, and costs. The realization of precision medicine in oncology could have a substantial impact. An estimated half-million cancer patients may be eligible for guideline-recommended targeted therapies each year and could benefit from demonstrated benefits of targeted therapy, including delay in tumor progression, longer survival, more quality-adjusted life years, avoidance of non-effective treatment, and lower treatment costs [2–8]. The FDA has approved > 90 pharmacogenomic drugs in cancer, and the National Comprehensive Cancer Network (NCCN) recommends precision medicine not just as a general approach, but for specific treatment decisions across a number of cancers, including breast, lung, colorectal, and melanoma skin cancer, among others [9–13].

Despite the guideline recommendations and the immense promise, not all patients who could benefit from targeted cancer therapy receive it and some who may not benefit actually receive it [14–16]. Only half of white elderly women and 40% of black elderly women with non-metastatic human epidermal growth factor receptor 2 (HER2)-positive breast cancer receive appropriate monoclonal antibody therapy [17]. Only 18% of colorectal cancer patients in the last decade with Kirsten rat sarcoma viral oncogene homolog (*KRAS*) wild-type tumors received anti-epidermal growth factor receptor (EGFR) antibodies [18]. Less than half of non-small cell lung cancer patients eligible for EGFR inhibitors receive them [19]. Although increasing in recent years, one-quarter of advanced lung cancer patients still do not receive tyrosine kinase inhibitors (TKIs) [20]. Furthermore, some cancer patients may be treated with targeted therapy where it is not warranted [21, 22].

Barriers to providers’ appropriate use of targeted therapies include knowledge and skill deficits [23, 24] and environmental and resource constraints, including lack of reimbursement, limited access to testing technology and treatments, and lack of time for the burdensome coordination of testing, therapy, and required follow-up [14, 25–34]. Technology limitations have also been recognized: test processing time often exceeds the treatment decision-making interval [35–37] and the specimens required for testing are difficult to obtain in some cancers [38]. Less studied are the motivational determinants that may facilitate or inhibit physician use of targeted therapy, although perceptions of limited utility and lack of patient receptivity have been noted as barriers for some genomic tests [36]. Recent policy-level changes and continuing evolution of the technology are lessening the impact of reimbursement barriers and testing accessibility [39, 40]. Training programs have been put in place to address knowledge deficits [24, 30]. These changes in the precision medicine landscape put into focus the need to explore the motivational barriers that oncologists and their teams may experience (e.g., beliefs about consequences and capabilities, social and professional roles and identities). Recent changes also engender the need to unpack the organizational contexts in which targeted therapy is delivered, particularly in the community oncology setting, because most cancer care in the USA is delivered in community practice. Despite this, few previous studies of precision medicine implementation have included US community-practicing oncologists among their samples [41–43]. The specific roles that community oncology teams play and the subsequent behaviors they perform in delivering targeted therapy are poorly understood. Furthermore, most interventions to improve precision medicine delivery have been focused on tumor boards or EPIC-based decision support tools, interventions which may have limited availability outside of academic settings [28, 42, 44–46].

Although identifying the most salient barriers can increase the likelihood that interventions are effective and changes are sustained [47–50], no existing studies have taken a comprehensive approach, using a theoretically derived implementation science determinant framework, to identify effective intervention points for implementing targeted therapy [32, 33, 46]. Furthermore, existing research does not specify the behaviors and delivery processes currently in place to pinpoint what changes need to occur.

We sought to use an established implementation science framework to identity actionable determinants of targeted cancer therapy use among community oncology practices and conduct generalized process mapping to identify current state processes across sites. We anticipate these results will be foundational to the development of implementation strategies to support guideline-based targeted therapy delivery in community oncology practice.

## Methods

We conducted a modified template analysis [51] of semi-structured qualitative interviews based on the Theoretical Domains Framework [52] and process mapping using the Rummler-Brache approach [53, 54]. The study was conducted under a protocol approved by the University of Kansas Institutional Review Board and is reported according to Consolidated Criteria for Reporting Qualitative Research (COREQ) [55].

Oncology care providers involved in the delivery of targeted therapy and practicing in community settings were eligible to participate and could include medical oncologists, surgeons, pharmacists, pathologists, healthcare administrators, nursing staff, or other ancillary providers, as we anticipated multiple healthcare professionals to be involved in such complex care delivery. We excluded academic providers and solo providers. Initial efforts were to restrict participation to providers practicing in a 13-state region in the Central USA and whose institutions were willing to participate in a companion medical record abstraction study, but due to COVID-19 pandemic-related practice disruptions, we altered the protocol [56] to expand recruitment by extending geographic reach to other US states and relaxing the requirement to participate in the medical record abstraction. We mailed invitation letters, signed by regionally prominent physicians, to all oncologists who billed Medicare in January 2020 in the 13-state region, inviting them to participate in a mixed methods study. We also targeted US pathologists and critical access hospital administrators via email and extended personal invitations to NCI Community Oncology Research Program principal investigators and administrators. Lists were obtained from the Centers for Medicare and Medicaid; Medical Marketing Service, Inc.; a National Rural Health Association Consulting Service, and directories on public websites [57, 58]. Once we identified an index provider within practices, we used a snowball sampling strategy to identify other care team members and allowed the index provider to specify the roles important at his or her institution around targeted therapy. No exclusions on provider roles were applied by the study team.

We used a semi-structured interview guide based on the Theoretical Domains Framework (TDF) [49, 59] to identify capability, opportunity, and motivational constructs key to targeted therapy use and allowed the interviewers to tailor questions to the interviewee’s role and involvement in targeted therapy delivery. The interview guide was modeled on previous determinants assessments conducted by our team [60] and relied on broad open-ended questions to identify many possible determinant domains, but encouraged probing on specific domains. To carefully and comprehensively specify the behavior, we used process mapping techniques in which we devoted interview time to detailing the targeted therapy delivery process, using a specific item to elicit process characteristics [61]. We collected or derived demographic information about sites from publicly available data sources, including the CMS Compare file, census information, Health Resources and Services Administration, the Kaiser Family Foundation, and the American Hospital Directory [62–65].

A single interview with each participant was conducted between July 2020 and May 2021. All interviews were conducted via telephone or video conference per participants’ preference. Verbal consent was obtained. Participants were offered a gift card for participation. Two female, PhD-trained qualitative researchers (SDE and JVB), a sociologist, and an anthropologist with > 30 years of combined ethnographic interviewing experience, but naïve to targeted cancer therapy delivery, conducted all interviews. The use of naïve interviewers was a pragmatic decision to align the skills of the study team with the study design, but interviewer naivete was used strategically to establish the interviewee as an expert and to elicit mundane details about targeted therapy delivery by making the process “strange,” in accordance with common ethnographic interviewing techniques [66]. A single practice interview was conducted and discussed to familiarize the team with the interview guide and key concepts related to precision medicine. Participants were unknown to the interviewers prior to the study interaction. Interviewers had no investment or biases toward targeted cancer delivery but were motivated to identify implementation strategies potentially effective for future studies. Interviews, ranging from 27 to 63 min, were recorded and interviewers collected brief field notes on each interview to assess the saturation and identify issues for follow-up in subsequent interviews.

Interview audio content was transcribed verbatim and coded in NVivo [67]. Coding consisted of assigning excerpts to defined TDF constructs described in a code book developed a priori, combined with inductive coding of (1) specific behaviors performed in the delivery of targeted therapy and (2) potentially distinguishing contextual factors informants reported. Interviewers kept memos during coding, and the study team met regularly to discuss the findings. In the initial analysis, two investigators (SDE and JVB) identified distinct behaviors and then attributed determinants to specific behaviors. After the initial coding, a third investigator experienced in the TDF framework (EM) reviewed all transcripts to ensure TDF constructs were consistently identified. Sub-themes were then identified within domains and key quotes displayed. Concurrently, we summarized all process descriptions into a single Rummler-Brache diagram, also known as a swimlane diagram [53, 54]. Based on descriptions gathered from participants, we identified the roles responsible for each part of the targeted therapy delivery process, represented by a single “lane” in the process map. We then summarized the targeted cancer therapy delivery process across all cancer programs, with arrows indicating handoffs across roles and diamonds representing decision points. We used distinct colors to represent contextual differences in processes or teams across sites. As part of iterative analysis, we identified pathologists as having important roles in targeted therapy delivery and sought additional input from pathologists to reach saturation. Across both the determinant coding and swimlane diagram, we noted contextual determinants reported to impact testing and treatment behaviors and summarized these factors by major domains.

Interviews and results were not returned to study participants for review but were shared at multiple time points with other community oncologists participating in a Cancer Center Disease Working Group. Feedback was used to shape interpretation and validate findings.

## Results

### Participant characteristics

**Fig. 1:**
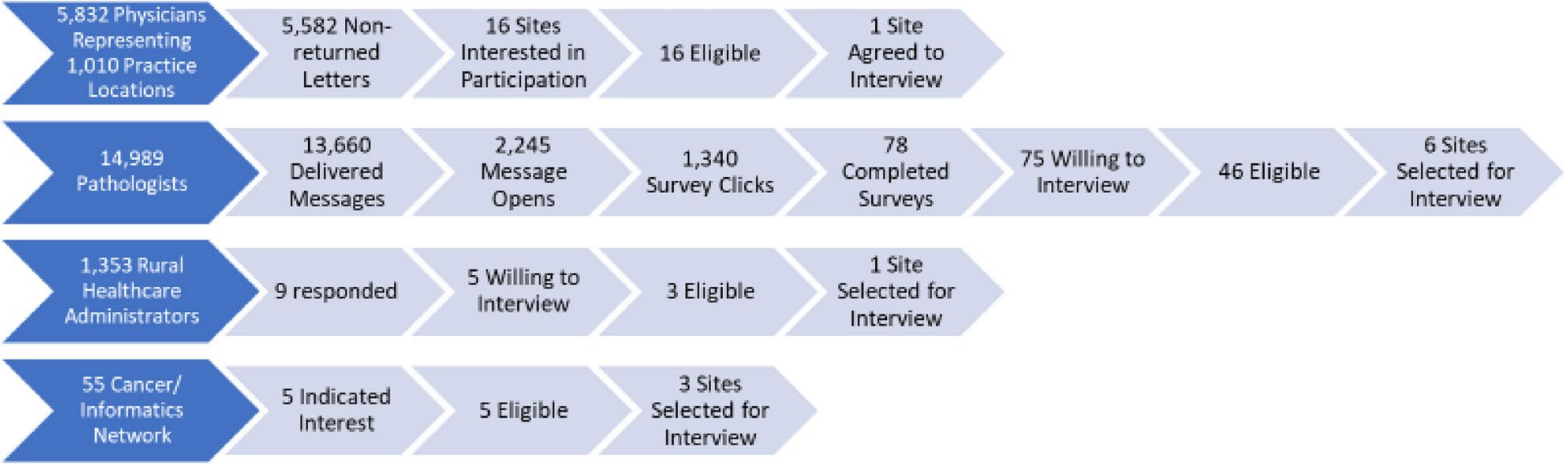
Study opportunity dissemination, interest, eligibility, and participation

Broad notifications about the study were pushed to 22,229 medical oncologists, pathologists, rural healthcare administrators, and research network personnel, which represented approximately 5013 non-unique practice contacts (Fig. 1). Across these notifications, 108 providers indicated interest of which 70 were considered eligible to participate. From this group, 24 individuals agreed to and completed the individual interview (range of 1–4 respondents/site). Individual participants represented a variety of roles in their cancer programs, including medical oncologists, pathologists, surgeons, pharmacists, healthcare executives, advanced practice nurses, and oncology nursing staff (Table 1). Ten participants held some type of leadership role within their oncology program or organization. Two participants had specialized training in genomics. Participants represented 11 community oncology programs, ranging in size, geography, and location. Table 2 describes the characteristics of cancer programs represented in the sample.

**Table 1:** Demographic characteristics of participants

	**Physicians (*N* = 12)**	**Non-physicians (*N* = 12)**
**Gender**		
Male	8	3
Female	4	9
**Professional role**		
Medical oncologist	5	
Pathologist	6	
Surgeon	1	
Pharmacist		4
Nurse or advanced practice nurse		5
Administration		2
Molecular geneticist		1
**Specialized training in genomics**	1	1
**Organizational leadership role**	4	6

**Table 2:** Characteristics of participating community oncology programs

	**Count (%) (*n* = 11)**
**Region**	
West	2 (18%)
Midwest	7 (64%)
South	2 (18%)
Northeast	0 (0%)
**Rurality**	
Rural	3 (27%)
Non-rural	8 (73%)
**Medicaid expansion state**	7 (64%)
**Critical access hospital**	1 (9%)
**Bed size**	
Small (1–49 staffed beds)	1 (9%)
Medium (50–99 staffed beds)	0 (0%)
Large (100–199 staffed beds)	2 (19%)
Very Large (≥ 200 staffed beds)	8 (72%)

### Behavior specification

**Fig. 2:**
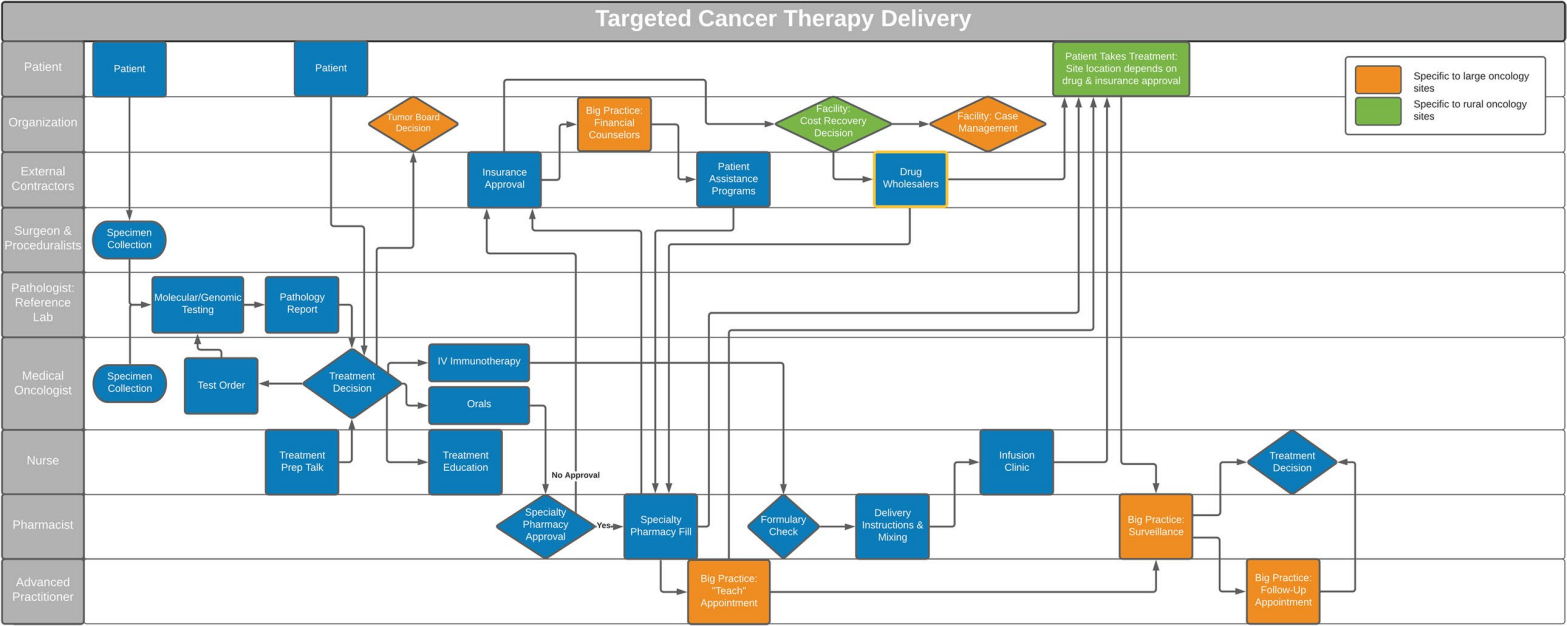
Swim lane diagram representing team interactions and processes across 11 community oncology sites

In eliciting the nature of the care delivery behavior from participants, we recognized two distinct behaviors across sites—testing and treatment decision-making—which were essential to targeted cancer therapy delivery. While successful implementation of targeted therapy requires both behaviors, each behavior consists of its own set of steps, and the behaviors are typically performed by different members of the healthcare team. The importance of the distinction was made more evident after we mapped the dual-behavior process onto a swimlane diagram (Fig. 2), as the inter-team interactions involved in each behavior were different: pathology-oncology teams interact in testing and pharmacy-oncology teams interact in treatment. As a result of process mapping, the study team recognized that pathologists were under-represented in the interview sample, thus additional pathologists were recruited until saturation was reached.

*Testing* processes were characterized by a bottleneck at some sites, leading to delays in treatment initiation, additional work by nursing staff, and anxiety for patients and their providers. At most sites, oncologists took a lead role in ordering molecular and genomic tests. Patients presented for treatment decisions after tumor biopsy and then oncologists ordered necessary tests. This sequence of events resulted in two potential treatment scenarios: either prioritizing expediency of treatment by proceeding with non-targeted therapy while awaiting testing results or prioritizing comprehensiveness by delaying treatment while awaiting final test results. The nursing staff at some sites had the role of managing the test results and coordinating the timing of patient scheduling to align. At other sites, the pathology team initiated molecular and genomic test orders, negotiated test reimbursement, managed the results, and shortened the window of time from diagnosis to treatment decision-making.

*Treatment* processes differed depending on whether the prescribed targeted therapy was delivered orally or infused. Importantly, because payor reimbursement policies differ, and costs and economic benefits accrue differently based on the mode of delivery, different care delivery teams within sites and different care delivery processes were used to execute targeted therapy delivery. Some community oncology programs did not have the organizational capacity to deliver both oral and infusion therapy. For example, some sites had nursing and administrative processes in place to deliver infused drugs but were constrained in their ability to access some treatments or to manage complicated payment programs to provide access to oral therapies for un- or underinsured patients. The size of the oncology program seemed to create variations in roles (Table 3). For example, nurse practitioners and pharmacists were involved in the delivery and management of targeted therapy at larger organizations. There was also variation among cancer programs in engagement with external organizations who could manage drug acquisition and reimbursement program management.

**Table 3:** Contextual factors potentially unique to small and rural cancer programs

Program characteristic	Domain	Testing	Treatment
**Small program size**	*Motivation*	• Smaller cancer programs were cognizant of the volume required to cover fixed costs of testing, in contrast to providers at larger programs	• Smaller sites expressed more concerns about their ability to deliver targeted therapy, acknowledging institutional costs and staffing limitations
	*Capability*		• Fewer staff have expertise in targeted therapy
	*Opportunity*	• May not have the volume required to cover fixed costs of in-house testing	• Have staffing limitations compared to larger organizations with a greater number of provider types (e.g., nurse practitioners and pharmacists) available to help with the delivery and management of targeted therapy • Perceived greater pre-authorization burdens • Experienced more limitations from drug wholesalers • Had fewer reserves to absorb non-reimbursed care • Could not have in-house specialty pharmacies to draw on for drug acquisition • Lacked navigation programs to help patients manage the complexity of targeted therapy • Lacked tumor boards • Lacked staff to deploy to facilitate reimbursement for targeted therapies • Lacked staff for “teach” appointments to facilitate adherence to oral targeted therapy and surveillance appointments
Rural	*Motivation*	• Saw great potential advantage of liquid biopsy over tissue testing to address unique delivery needs	
	*Opportunity*		• Hospital has invested in infusion center as a way to increase revenue • “It’s getting harder and harder to find RNs. We have several openings right now for RNs. We’ve had a lab position open for two years. We have to use an agency.” [Rural Administrator] • Facility lies outside of the shipping zone for targeted therapy

### Behavioral determinants

Variation in the successful implementation of targeted therapy across sites was reported. Because we identified different determinants and actors for testing and treatment, which have not been distinguished in prior determinant studies, we present motivation, opportunity, and capability determinants for each behavior separately. Additional file 1: Tables S1, S2, and S3 provide exemplary quotes across each TDF domain.

#### Motivational domains

The intention to perform molecular and genomic testing and to provide targeted therapy to eligible patients was prevalent and strong across all interviews (Additional file 1: Table S1). Providers acknowledged the high demand from providers and patients for testing and characterized targeted therapy as “the way of the future” [rural oncologist]. For *testing*, providers perceived mostly positive consequences, including providing something of benefit to patients and to themselves. They believed molecular and genomic testing (and College of American Pathologists (CAP) protocols) helped them to meet professional standards and avoid audit failures. Although participants expressed concerns about patients’ potential out-of-pocket costs, which created negative reinforcement, some perceived societal benefit as they considered the (low) cost of testing relative to the (high) cost of unnecessary therapy.

*We’re talking about tests that cost about $5000 maybe and in terms of the cancer center or cancer patients’* [United States] *costs of their disease therapy, it’s a drop in the bucket and why not have the best information available? To me if I had something and I wanted to have it treated, I would want to know what I’m treating.* [Non-rural Pathologist]

Others limited cost concerns to tests that have no actionable treatments; still, others acknowledged broadening reimbursement for testing. Beliefs about the limited capability to acquire sufficient tissue for testing were widely acknowledged, and enthusiasm was dulled by the rarity of actionable mutations.

We found wide variation in role assignment for genomic test ordering across community oncology programs that seemed to impact guideline adherence and timeliness. In addition, these roles were in flux. Both oncologists and pathologists noted that their roles were changing because of targeted therapy, sometimes creating communication failures across teams that impact task proficiency. Some programs relied on oncologists to order somatic tests, whereas other programs assigned the responsibility to the pathologist. Pathologists typically welcomed this role in subtyping tumors, acknowledging that genomic testing aligns with their existing responsibility to stage tumors.

*Ultimately, we are not only the stewards of the tissue but we’re also the owners of the classifications and it is not enough anymore to be good at recognizing things under a microscope. We do understand what is driving these diseases and the relevance and mechanisms that are altered or disorganized with each one of these translocations with the exception of maybe somebody doing clinicals and ethics, we are the physicians that are closest to what is happening and most pathologists have a research background and some kind of familiarity with molecular and genetics…So I think we’re going to be the driving force.* [Non-rural Pathologist]

Some sites created new roles for managing the multitude of reference lab orders, tracking test results, ensuring tests were incorporated into the electronic medical record and made available at the point of treatment decision-making, but other sites relied on physicians or other clinical staff to be responsible for this work. Consequently, they experienced delays in obtaining and reviewing test results, which created anxiety for both patients and providers. Many participants were highly motivated to identify strategies to facilitate interdisciplinary communication, and at least one program did so by creating new roles to manage inter-team communication needs, potentially alleviating providers’ fears and frustration surrounding test results interpretation.

…*they came up with this idea of having a pathology liaison attending the hematology oncology meetings and because I had a background in molecular research, I happened to be the one.* [Non-rural pathologist]

Some challenges to role realignment were acknowledged: inertia and industry marketing of tests to physicians. In addition, participants’ beliefs about their capabilities determined patterns of use. Smaller sites expressed more concerns in their ability to deliver targeted therapy, acknowledging institutional costs and staffing limitations. Smaller cancer programs were cognizant of the volume required to cover fixed costs of testing, in contrast to providers at larger programs:

*I’m* [at] *a large cancer hospital. I don’t even check what kind of insurance the patient has. I do the same thing for everybody because the hospital eats the cost if Medicare doesn’t reimburse.* [Non-rural Pathologist]

Rural providers saw the great potential advantage of liquid biopsy over tissue testing to address their unique delivery needs.

Providers had high intentions and motivation to use *targeted treatment*. Providers saw mostly positive benefits of targeted treatment, believing it provides more treatment options, higher response rate, better outcomes, more convenient delivery modes, fewer toxicities, and, consequently, fewer medication side effects than standard therapy. Observing these treatment benefits contributed strongly to adoption among participants. However, targeted therapies were not seen as exclusively beneficial. The high costs of the therapies were of concern (although participants acknowledged generous industry subsidies) as were the side effects patients experience. Although targeted therapies were perceived to have a relative advantage over standard therapy, some questioned whether the high costs were worth the benefits, as patients may have better survival or other outcomes, but remain without a cure. Furthermore, very few patients were eligible for the treatments. Echoing these concerns and the infancy of the field, several participants acknowledged that the full promise of precision oncology has not yet been realized.

Role conflicts were also apparent with targeted treatment delivery. Regarding their professional role and identity, community oncologists saw their role as changing dramatically with a greater need to subspecialize, a difficult transition for those established in practice.

*I’m a general medical oncologist. I probably know some areas better than others but I treat technically anybody who walks in the door with any type of cancer.* [Non-rural Oncologist]

[I]*t’s very, very hard to be a general oncologist anymore. The field has exploded with each cancer almost a unique specialty of its own so increasingly we will see doctors who only see one type of cancer because the field keeps changing.* [Rural Oncologist]

Beyond treatment decision-making, care delivery also involved monitoring side effects, treatment adherence, and disease progression, and this role was filled by different professions depending on the size and capacity of each cancer program.

#### Capability domains

For both testing and treatment, the pace of knowledge creation surpassed all providers’ ability to keep up, particularly because community oncologists practice as generalists, creating the need to keep abreast of advances in all cancer types (Additional file 1: Table S2 for themes and quotes). Because they so rarely see any one patient eligible for targeted therapy in their practice, maintaining current knowledge is difficult. Furthermore, biomarker discovery often outpaces actionable recommendations; thus, much information directed to them in the literature and in testing reports was not relevant for treatment, and sometimes required expertise they do not have to interpret. Testing required special communication skills, both with patients and with colleagues from different disciplines, to alleviate the perceived untenable knowledge requirements.

*We have a great working relationship with our pathology group. They’re very open to …change … based on NCCN guidelines and recommendations as to what they reflexively test for, so…docs aren’t the ones having to know all this stuff and constantly be ready to order ALK and ROS and EGFR. Some things now, if it’s a lung cancer patient, it reflexively is being performed and sent out by pathology. That’s also helped speed up the process…So reflexive testing and a relationship with your Pathology Department I think is key so that you’re getting those things.* [Non-rural Nurse]

NCCN guidelines were frequently mentioned across interviews as a strategy to promote proficiency, both for testing and for treatment. CAP protocols regulated behavior for many pathologists; pharmacists found board training materials important for informing treatment decisions. However useful, users saw opportunities to improve these materials to facilitate implementation, with disease-specific (rather than biomarker-specific) protocols for testing and standardized results reporting as additional needs. Although professional society guidance supported first-line testing and treatment decisions, more standardization of testing protocols, both institutionally, and within guidelines to accommodate second and subsequent treatment decisions were desired. Some cancer programs used pathways which embed guideline-concordant precision oncology into the electronic health record; others relied on tumor boards for enhancing knowledge deficits, both of which targeted physicians. Notably, very few participants reported auditing their testing or treatment performance, and the recognition of its absence was described only in the context of testing.

*Our data is growing thousands of patients, so we need someone to spend time to analyze that because if somebody analyzed those -- and I do that, but it’s just case by case because I’m the only one.* [Non-rural Pathologist]

#### Opportunity domains

The organizational and larger policy environments were not perceived as supportive of testing or therapy, underscoring perceived contextual differences in community oncology programs (Additional file 1: Table S3). For *treatment*, the cost of targeted therapy created individual and organizational work. Providers were aware of the substantial treatment costs and co-payments many patients face. They perceived the pharmaceutical assistance programs to be generous in providing drug assistance for patients who needed it, but participants described staff with roles and considerable responsibilities primarily dedicated to managing drug acquisition, rather than patient care. Only FDA-approved indications were reimbursed by the pharmaceutical assistance programs and payors, and because of the high cost, made off-label use costly to the organizations and to providers personally. Rural and smaller cancer programs had particular challenges in acquiring targeted therapies for their patients (Table 3). They perceived greater pre-authorization burdens, more limitations from their drug wholesalers, fewer reserves to absorb non-reimbursed care, and fewer staff with expertise in targeted therapy. Larger cancer programs had their own specialty pharmacies on which they could draw for drug acquisition. Navigation programs were seen as important to help patients with some of these challenges, but it was acknowledged that these resources were only available for certain cancer types, leaving other cancer patients’ needs unmet. The NCCN guidelines were not only used to regulate behavior, but also as a resource to understand what would be reimbursed. Surveillance of therapy, once it was acquired, was complicated for those on oral agents, as they had less scheduled interaction with nursing or pharmacy staff than those receiving infused therapies, creating concerns about adherence and side effects.

For *testing*, reimbursement was also a concern, dictating not only which test, but which testing platform could be used, and when testing could be ordered. Providers acknowledged that only actionable biomarkers were eligible for reimbursement. However, testing faced additional organizational constraints. Few community cancer programs had the capacity for in-house genomic testing; thus, most tests were sent out to reference laboratories, which were perceived by some to accrue a greater cost burden to them. Clinical trial enrollment and reflex testing (pathologist-initiated testing) were strategies used by some sites to mitigate reimbursement challenges and testing delays, but preauthorization requirements lessened the effectiveness of reflex testing. Molecular tumor boards were seen as very useful in identifying what to test and how to interpret tests. However molecular expertise was often lacking at community cancer programs, so industry resources were welcomed.

[Testing company] *will help any group coordinate a molecular tumor board and be on the call and help review those results. At any time, certainly we could continue our molecular tumor board without them but it’s …been great for us all to learn together and for their scientists sometimes to hear from the clinician perspective.* [Non-rural Nurse]

At least one site not only ran molecular tumor boards, but also integrated molecular specialists into existing disease-specific tumor boards.

*When I came* [here],* I tried to establish a molecular committee and it was really hard so instead of that we started attending the Hem Onc meetings.* [Non-rural pathologist]

This interaction in the disease-specific tumor boards was influential in creating shared understanding of the tests and treatments appropriate for patient care.

## Discussion

We interviewed a wide range of cancer care providers involved in the delivery of targeted cancer therapy in diverse community-based cancer programs, including those not typically included in precision oncology implementation research. Like previous studies of academic and international programs [68, 69], we found similar capability and opportunity constraints in community oncology programs. However, our study extends the existing literature by highlighting a larger range of motivational determinants that can facilitate but also slow implementation of targeted therapy and, potentially, other healthcare innovations. Leveraging these determinants may lessen large institutional investments in clinical decision support currently considered necessary to meet perceived physician capability deficits.

Across the sample, there was a steadfast intention to provide targeted therapy to cancer patients eligible for it, a finding recently replicated [69]. Nonetheless, our study documents differences in other motivational domains that may be important, namely concerns about the high cost–benefit ratio of treatment and role identification of the professionals involved in it. Testing is perceived to have societal benefits, allowing for stewardship of costly treatments, in addition to patient benefit. In contrast, the benefits of targeted therapies are not as universally regarded. Although they vastly improve outcomes for the few patients eligible for them, they do not cure disease, and costs per dose and per course are perceived to be high for both patients and for the institutions delivering them. These beliefs could influence perceptions of who should bear the cost of organizing coverage. Although currently pharmaceutical company treatment stipends are seen as offering the uninsured wider access to targeted therapy, they are cumbersome and contribute to the additional uncompensated work oncology programs must provide. Because pharmaceutical companies realize all the benefits by increasing their market share through these programs, they could potentially balance the lack of societal benefit by standardizing copayment programs across companies, make eligibility criteria explicit, and broaden qualifications.

To our knowledge, this study is the first to illuminate ambiguity about *who *should initiate genomic and molecular testing for cancer. Our study suggests that targeted therapy delivery is difficult because it requires incorporating the new task of genomic test ordering and interpretation into the work scope of professionals typically responsible for treatment decision-making, delivery, and monitoring [70]. Most cancer programs relied on oncologists to order somatic tests, the purpose of which is to fully stage the tumor to ensure treatment is appropriate for the patient. However, because the oncologists’ role is focused on treatment, the role of staging the tumormay be at odds with their typical responsibility. Whereas for pathologists, definitively staging a tumor falls within existing responsibilities [71]. It also aligns with their need to allocate scarce tissue optimally, making pathologist-centered implementation strategies very promising. Some sites had instituted pathologist-initiated test ordering for guideline-recommended tests. Called *reflex testing*, automatically ordering one or more secondary tests based on preset criteria applied to the initial test, has been demonstrated to have numerous benefits, including increasing testing rates [72] and identification of mutations or other molecular abnormalities [73, 74]; reducing unnecessary testing [75, 76], unnecessary care [77], disparities in care [78], and time to treatment [72, 79]; and improving outcomes [80] and healthcare operations [81]. It has been shown to be cost-effective [82] and to reduce costs [83, 84], mainly by focusing on testing for approved and clinically actionable molecular alterations. Future studies should consider the effectiveness of reflex testing to reduce role dissonance among the cancer care delivery team as well as its impact on patient outcomes.

Our findings further extend our understanding of motivational determinants in that few sites reported monitoring testing and targeted therapy use, making it unclear whether their efforts were successful or equitable. Most practices did not have the necessary measurement tools, staffing, or infrastructure to monitor their own performance and thus may not have the performance knowledge needed to regulate their behavior. A limited number of measures related to genomic or molecular testing and treatment are available, required by accreditation agencies, and routinely included in cancer registries [85, 86]; thus, support to develop and implement such monitoring at an institutional level may be needed. Sites used known strategies for improving individual knowledge and treatment decision support but lacked inter-team processes to standardize testing across the eligible patient population.

Similar to previous reports, community oncology program participants acknowledged knowledge and skill deficits in testing and treatment, especially given the rapid developments in the field [69, 87]. Awareness of targeted testing and treatment among our sample appeared high, but participants were less confident in their “how-to knowledge” or their ability to apply appropriate knowledge about testing and treatment options in practice [87]. However, rather than advocate for more education to fill knowledge gaps, an implementation strategy to which technology developers often default, participants in this study suggested institutional-level standardization of testing aligned with clinical practice guidelines, and results reporting and treatment education which prioritizes actionable mutations (i.e., only those mutations associated with existing evidence-based treatments) to overcome capability barriers. Others have characterized the actionability gaps in precision medicine [69] and called for research to enhance clinical utility [88]. Our findings suggest that treatment decision-makers prefer prioritization of actionable mutations in results reporting, consistent with existing reporting guidelines [89, 90]. Thus, opportunities to improve result communication, consistent with previous research [91–93], remain.

Also similar to prior research, our findings highlight significant opportunity barriers to targeted therapy use, namely the high cost of both testing and treatment, that have long been perceived as implementation barriers [41, 94, 95]. However, our findings also reflect recent transitions in reimbursement which decrease patients’ out-of-pocket expenses for testing [39, 71, 96], and assign responsibility for billing to pathology laboratories, shifting incentives for testing from physicians to hospital cost centers [39], and creating new organizational landscapes. In addition, we found that most community cancer programs have made organizational and personnel changes to ensure the delivery of costly targeted therapy by repurposing highly skilled oncology nurses and pharmacists to manage complicated and time-consuming payor and industry requirements. Some cancer programs designed new organizational units to efficiently manage genomic test procurement, tracking, and reporting. Unlike organizational changes to testing management, whose efficiencies may benefit the organization, the addition of new reimbursement and treatment acquisition roles required to deliver targeted therapy to un- and under-insured patients, are not costs that can be recouped. Aside from potential reputational prestige, healthcare organizations bear the cost of these activities with little direct benefit.

The fixed cost of these new roles, no matter how streamlined, can only be borne by practices with high volume. Likewise, specialized services, such as specialty pharmacies, which can be revenue-generating for an organization, are not feasible among low-volume sites, potentially creating disparities among smaller community practices. Some rural hospitals have created infusion centers to build sustainable revenue and may be threatened by targeted therapies which can be administered orally, bypassing their billable infusion services. Both smaller and rural programs contrasted their capabilities and the disproportionate impact of organizational and policy decisions. Because genomic testing occurs in reference laboratories, outside of most community practices, we noted fewer testing differences than treatment differences among community practices, suggesting that test-ordering interventions may be more feasible than treatment interventions which will require substantial addition of resources to address. Furthermore, ensuring appropriate testing at smaller and rural community practices could potentially facilitate care when patients are referred for treatment at larger community or academic practices. Nonetheless, the evaluation of precision oncology implementation strategies should assess differential effectiveness among small and rural cancer programs to ensure benefits are realized equitably.

Finally, our description of the process from multiple team members’ perspectives, specification of testing and treatment as two distinct behaviors, and comprehensive elicitation of all motivational domains adds new understanding of the strong facilitators and unique barriers community providers experience. In particular, our identification of how determinants of testing behavior differ from the determinants of treatment behavior is a unique contribution not only to understanding precision medicine implementation, but also to the field of Implementation Science. By contrasting the determinants of testing with those of treatment, we uncovered unique patterns of determinants and opportunities to respond to areas of significant delay. Others have distinguished testing as a process outcome separate from precision medicine application [68]. However, specifying testing and treatment as two separate behaviors, each with their own determinants, allows us to consider the different teams involved and connect efforts currently siloed in the fields of pathology and oncology. Although careful specification of *implementation strategies *is widely encouraged across the field [97–99], less emphasis has been placed on the careful specification of *intervention behaviors *in assessing the behavioral determinants which the implementation strategies are designed to overcome. Instead, most frameworks emphasize understanding the contexts in which innovations are implemented. For example, the Consolidated Framework for Implementation Research (CFIR) emphasizes adapting for context as the key to successful implementation, rather than a thorough specification of the behavior to be changed [100]. CFIR is not unique; the relevance of context is pervasive throughout implementation determinant frameworks [101]. In a survey validation of domains identified in the TDF, the *Nature of the Behavior* construct was dropped from the Framework as it aligned statistically as a separate task, apart from other determinants [49]. Although Cane, O’Connor, and Michie adamantly emphasized that understanding the nature of behaviors is key to analyzing implementation and other behavior change, they removed the construct from the TDF. Instead, they included behavior specification as one of the 8 steps in intervention design in their complementary behavior change wheel (BCW) approach [49]. Influenced by the BCW in a previous study in which we identified a promising implementation strategy [102, 103], we subsequently have used careful specification of complex cancer care delivery behaviors to uncover previously unreported determinants [104], including in this study. Our use of process mapping, particularly the swimlane diagram [53, 54], as a tool to specify the behavior may be a unique contribution but should be tested as a potentially fruitful addition to determinant analysis.

### Limitations

Our use of a qualitative study design over a quantitative design allowed us to identify new motivational domains that are a key barrier to genomic testing. However, the initial narrow geographic focus of recruitment and requirement to share quality measures, as well as the use of snowball sampling and the small sample inherent to the qualitative study design, holds *potential* to limit the generalizability of our findings. Secondly, although we presented our findings to community oncologists and shaped our interpretation by their reactions, we did not formally conduct member checking [105] to ensure the credibility of results with participants in this study. Subsequent evidence suggests that the threat to the validity of these design decision may be low. We subsequently conducted a national survey of US pathologists (unpublished) which confirmed barriers to genomic testing and preferred solutions similar to those reported in this study. A recent study of oncology care teams [69] conducted in Australia confirms our findings of high motivation to use targeted therapy in the current era, suggesting differences in motivation between our study and earlier studies may reflect changes in trends over time, rather than groups of providers who hold discordant views.

Our study was designed to elicit barriers and best practices related to somatic alterations in tumor tissue. It was not intended to elicit barriers to genetic testing for inherited risk. Hereditary testing typically informs a patients’ prognosis, or risk of disease, whereas somatic alterations arising in the tumor can determine whether a treatment will be effective or not. Although some hereditary testing has received FDA approval for treatment decisions, we did not focus on germline testing. We understood physicians to perceive these two types of tests to have different utilities. But because there remains confusion between prognostic and predictive testing and blurring in FDA-approved uses, additional research to assess understanding of, and concerns about, these two types of tests are warranted. Finally, our study was designed to comprehensively elicit a broad range of barriers and best practices but was not designed to draw comparisons between urban and rural programs or large and small programs. Thus, future research should validate the differences we observed among a larger sample of programs. Similarly, the salience of each construct was not evaluated. Future surveys using representative sampling could narrow these constructs to those deemed most important to community oncologists, but implementation strategies matched to identified determinants should be compared in prospective trials.

## Conclusions

Cancer care providers view precision oncology as the wave of the future but our study identifies several motivational challenges that could potentially be addressed through role re-alignment. In addition, opportunity determinants may differentially impact small and rural sites. Standardized pathology-initiated genomic testing may prove fruitful in ensuring patients eligible for targeted therapy are identified, even if the care they need cannot be delivered at these sites. Finally, behavior specification may need to be explicitly and routinely included in the determinant analysis to identify the most promising implementation strategies.

## Supplementary Information

**Additional file 1: Table S1.** Motivation Determinants for Testing and Treatment. **Table S2.** Capability Determinants of Testing and Treatment. **Table S3.** Opportunity Determinants for Testing and Treatment.

## Data Availability

The datasets generated during the current study are not publicly available due to the potential breach of privacy by the small number of participants recruited but are available from the corresponding author upon reasonable request.

## Abbreviations

EGFR: Anti-epidermal growth factor receptor
BCW: Behavior change wheel
CAP: College of American Pathologists
COREQ: Consolidated Criteria for Reporting Qualitative Research
CFIR: Consolidated Framework for Implementation Research
EM: Emily Morrow
HER2: Human epidermal growth factor receptor 2
JVB: Joanna Veazey Brooks
*KRAS*: Kirsten rat sarcoma viral oncogene homolog
NCCN: National Comprehensive Cancer Network
SDE: Shellie D. Ellis
TDF: Theoretical Domains Framework
TKI: Tyrosine kinase inhibitors
