# Supplementary material for: Determinants of targeted cancer therapy use in community oncology practice: a qualitative study using the Theoretical Domains Framework and Rummler-Brache process mapping
Source: Implement Sci Commun. 2023 Jun 12;4:66. doi: 10.1186/s43058-023-00441-3 (PMC10259814; doi:10.1186/s43058-023-00441-3)
Supplement: Supplementary file 1 — Additional file 1: Table S1. Motivation Determinants for Testing and Treatment. Table S2. Capability Determinants of Testing and Treatment. Table S3. Opportunity Determinants for Testing and Treatment. [file 43058_2023_441_MOESM1_ESM.docx]

**Supplemental Table 1. Motivation Determinants for Testing and Treatment**

| **TDF Construct** | **Testing Themes** | **Treatment Themes** |
| --- | --- | --- |
| **Intentions**  *A conscious decision to perform a behavior or a resolve to act in a certain way* | **Patient and Providers Want to Test**  *I think all of the patients and all of the providers want to send these tests off* [Non-rural Oncologist]  *Dr. (name) had really been pushing for it. That’s something he’s very passionate about. He really believes that cancer patients are going to want to know that they’ve been screened…* [Rural Administrator] | **Targeted therapy is standard practice**  *I don’t think we can NOT do it. It’s just the way of oncology and how it’s proceeding and I think that we have an obligation to our patients to give them everything that’s out there.* [Non-rural Oncologist]  *We always look for the targeted treatment, the options. Those who have the targets that would be the first option.* [Rural Oncologist] |
| **Beliefs about consequences**  *Acceptance of the truth, reality,* *or validity about outcomes of a* *behavior in a given situation* | **Patients benefit from testing**  *...it is worth it in that* […] *when you get those results back and patients have a mutation where they can go on a…targeted drug…it’s totally worth it to me to know that information and the patients be able to benefit from that.* [Non-rural Nurse]  **Cost of test small relative to treatment**  *We’re talking about tests that cost about $5000 maybe and in terms of the cancer center or cancer patients’ United States costs of their disease therapy it’s a drop in the bucket and why not have the best information available? To me if I had something and I wanted to have it treated, I would want to know what I’m treating.* [Non-rural Pathologist]  **Concern about scope and cost of testing (how narrow or broad to order)**  *Certain oncologists will run everything under the sun whereas other oncologists will say no,* […] *that’s going to cost a million dollars, I’ll just focus on these few that I know I’m going to be able to treat whereas others don’t think of it that way.* [Non-rural Pathologist]  **Results may not be beneficial as patients expect**  *I think it’s critical* […] *to kind of manage patient expectations along the way because often times these are people in very desperate circumstances and you just want to make sure that they understand [testing] may or may not be really beneficial for them.* [Non-rural Physician]  *Other barriers are just the number of patients who don’t have an actual mutation.* [Non-rural Oncologist]  **Patients will get a large bill**  *the insurance company who then decides we’re not going to pay for this and then the patient gets those $10,000 molecular pathology bills and are the patients even aware that this tissue is being sent out for these kind of targeted, you know, looking for these different molecular targets?* [Non-rural Pathologist]  **Reimbursement is getting better**  *Insurance coverage is a little bit of an issue so getting the next generation testing is not universally covered. I think it will be more and more covered I think as it’s incorporated more into the guidelines that will happen more.* [Non-rural Oncologist] | **More treatment options**  *It’s given patients certainly treatment options that they didn’t used to have in terms of, as I tell patients, your ‘options in the pot’. There now tends to be more options in the pot generally speaking.* [Rural Nurse]  **Better Outcomes, Fewer Toxicities**  *It’s shown in most cases that if you add this on and you target that specific mutation that you’re providing better care. You’re providing more survival.* [Non-rural Pharmacist]  *In general the targeted therapy has a higher response rate.* [Rural Oncologist]  *Rather than kind of using the slash and burn approach which has dominated chemotherapy for the last sixty/seventy years, targeted therapy seems to in most cases give patients better outcomes but way less toxicities which I think has really benefitted everyone.* [Non-rural Pharmacist]  **Delivery of treatment is easier for patient**  *… patients don’t have to have a line placed. They don’t have to go into clinic once a week or even every day for a period of time and get admitted to the hospital for getting chemotherapy ...*[Non-rural Pharmacist]  *…doesn’t make them take a day off work or whatever to come get a treatment.* [Rural Nurse]  **Some see different side effects, not fewer**  *That is not to say that there are no side effects and in fact sometimes you will see some unique side effects which can limit treatment. Some patients cannot tolerate it.* [Rural Oncologist]  **Lots of promise, not all actualized**  *I’m probably not alone in saying that targeted therapy has enormous promise. but I think it is still probably I’d say sort of in the advanced infancy phase.* [Non-rural Oncologist]  **Cost worth the benefit?**  *…the irony is there is no targeted treatment that is going to cure you. You would think for the price that you’re paying that you should get a cure but unfortunately it’s not.* […] *Targeted treatment most often is an ongoing treatment.* [Rural Oncologist] |
| **Beliefs about Capabilities**  *Acceptance of the truth, reality* *or validity about an ability, talent,* *or facility that a person can put* *to constructive use* | **Reimbursement considerations differ at large vs. small centers**  *We’ve got to realize any lab tests we run, there has to be sufficient volume to justify paying for the quality controls and all the stuff. If you run one test or 20 tests, you still have to do the quality controls on that particular test.* [Rural Administrator]  *I’m [at] a large cancer hospital. I don’t even check what kind of insurance the patient has. I do the same thing for everybody because the hospital eats the cost if Medicare doesn’t reimburse.* [Non-rural Pathologist]  **(Not enough) tissue is the issue**  *Well, the main concern, number one, getting adequate amount of tissues from the biopsy so far is the biggest challenge. I’m sure it’s a universal problem because I’ve heard it from a lot of my colleagues too.* [Non-rural Physician]  *…half the time we do not have enough samples to even run the basic of the molecular study. Forget about extending a panel.* [Rural Physician]  **Liquid biopsy may be good for rural patients**  *I’m not in a big city, […] send the patient to [City], […] it’s complicated. You have many social issues, transportation, so sending a liquid biopsy, boom. That’s just a big solution to us, so I’m using that more and more as well*. [Rural Physician]  **Delays create challenges**  *[The] number 2 challenging is the waiting time to get a molecular panel result back.* [Rural Pathologist]  *It’s hard to know how long it takes. Sometimes those results – and they just trickle…well if you order twelve tests from them because it’s a panel…but you get 5 results. They don’t tell you that 5 more are pending so then you’re like okay, I have them all. […] on the paper it doesn’t tell you you have 5 of the 10 results.* [Rural Nurse] | **Cost is prohibitive to small infusion centers**  *Rural hospitals…they don’t have that much volume, they have to...when [patients] cannot afford it and straight off the bat that excludes medications such as immunotherapies which is so expensive…*[Rural Oncologist]  **Sometimes targeted therapy is more easily reimbursed**  *My nurse who is there, she is very good at calling them and getting it approved but it goes to a specialty pharmacy. …if there is anything to do with insurance, they run it and it’s been covered. So the access to targeted treatment paradoxically in the outreach clinic is not going to be a problem because like I said, it’s a mail-order prescription. It’s a pill. It is the IV treatments which are a problem. So immunotherapy the problem with immunotherapy it’s IV treatment so immediately that’s a hospital problem whereas a pill is more a patient problem, like adequate insurance and things like that. So targeted treatment is ideal for rural settings because it takes the responsibility off of the hospital.* [Rural Physician] |
| **Emotion**  *A complex reaction pattern, involving experiential, behavioral, and physiological elements, by which the individual attempts to deal with a personally significant matter or event* | **Delays create anxiety**  *I would say that the other thing is time to get the results…The delay is often probably like 7 to 10 business days so it can be like 2 weeks and again, when people are terminal and when they have a pause in their treatment it causes a lot of anxiety*. [Non-rural Oncologist]  **Heterogeneity in Reporting is Frustrating**  *The scattered nature of different genetic testing and that integration into your health system EMR I mean is frankly miserable at least here. There are multiple different tests for multiple companies. They’re all scanned PDFs. That’s exceptionally frustrating and I think it really hinders its quick utilization and being able to effectively use the info.* [Non-rural Pharmacist]  **Targeted therapy knowledge is overwhelming**  *A little bit of fear about: knowledge is accumulating rapidly and I don’t understand it but I’m being asked to use it or I know I should use it but I don’t understand it.* [Non-rural Surgeon]  **Molecular pathologist relieves fear**  *I think it’s super helpful to have a molecular pathologist. People are afraid of what they’re not familiar with and so having a molecular pathologist who is a bridge to the other pathologists can help overcome some of the resistance.* [Non-rural Pathologist] | **Anxiety changes treatment**  *Patients get super anxious too and they want to be started on something and they don’t want to have to keep waiting on, they don’t want to have to wait and wait and wait on results although most of the time theoretically another two week delay isn’t going to be a major game changer but to the person, it certainly feels that way.* [Non-rural Nurse]  **New drugs create fear**  *My biggest fear is again do I have enough knowledge and does my staff have enough knowledge because the phone calls, for instance as we speak, people are having side effects. They’re calling my nurse in clinic. She’s the first point of contact. She’s got to understand. She knows they’re on the drug but she’s got to be able to assess how serious is this side effect and what do I do? If we only have one person on the drug and it’s pretty darn new and nobody else in the clinic has anybody else on the drug, she’s got to have a resource. I’ve got to have a resource.* [Non-rural Physician]  **Excitement about new treatments**  *For the most part, it’s exciting.* […] *It’s exciting to find that there’s new things out there that is actually working for treatments that weren’t available years ago when we started this. You used to have to have six hours of chemo treatment and now you can get it in a pill that you take once a day. That’s exciting. They’ve made progresses, and the treatments are more tolerable…so that’s the exciting part is you actually see something that it feels like the research is starting to pay off; that something is actually there and helping the patient.* [Rural Nurse] |
| **Motivations and Goals**  *Mental representations of outcomes or end states that an individual wants to achieve* | **Make process easier by interdisciplinary communication**  *So it’s worth it. We’ve got to figure out a way to continue to be able to do it and a way to just make the process easier* [Non-rural Nurse]  *So, it’s a situation that where I think best practice is one that you have a good and frequent communication with your pathologist about what mutations we’re actually looking for and how that’s constantly evolving from various diseases.* [Non-rural Administrator] | **To be part of the research**  *Other motivators are contributing to the literature that’s coming out and contributing to studies. Depending on the cancer type and where they are, NCCN recommends looking for a study for some of these patients because that’s what will help the most people in the end.* [Non-rural Pharmacist]  **Get patient costs covered**  *You don’t want cost to be the one deterring factor for somebody to get treatment for their cancer. I think that’s not really fair that you hit the genetic un-jackpot. You’ve got cancer. Cost shouldn’t be the deterring factor for why you have to walk away from a medication that could add months more so even years to your life and so I think we’ll try, we’re going to fight our hardest here to try and get medication for every patient as much as possible.* [Non-rural Pharmacist] |
| **Reinforcement**  *Increasing the probability of a response by arranging a dependent relationship, or contingency, between the response and a given stimulus* | **Previous insurance denials create negative feedback**  *Because patients called us in the beginning and said “I got a bill for sixty-five hundred dollars, my insurance won’t pay for it”.* [Rural Nurse]  **Inertia limits change in ordering behavior**  *Well, I think that oncologists get very comfortable with a certain lab and assay. I think they’re marketed to very well, aggressively to use these certain tests, that they go to their conferences. They have reps visiting them and sending them information, and I think they would just rather keep up with what they’re doing and what they know. Their staff knows how to fill out the paperwork. It’s become a protocol or a routine, so I think it would take a lot to get them to change their behaviors. Now, if [hospital] would mandate them to use these, that would maybe be another thing, but I don’t think that the organization wants to alienate the oncologists or make them angry by forcing the issue, at least they haven’t so far.* [Non-rural Pathologist] | **Treatment depends on external contractors**  *In new drugs coming out, constantly the manufacturers are really looking at the specialty pharmacies and looking at the ratings on them. They’re looking at-everybody does patient surveys. They’re looking at that to see […] Is this someone we really want to go into a partnership with for our medication?* *[…] there are contracts between specialty pharmacies and insurance companies …insurances will partner with certain specialty pharmacies so not every patient can fill at every specialty pharmacy across the country. …Sometimes it gets a little tricky because if you’re dealing with what’s known as a limited distribution drug, so those are the ones where only certain pharmacies are contracted with the manufacturer to get the medication and then you’ve got only a certain number of pharmacies that are contracted with an insurance.* [Non-rural Pharmacist] |
| **Social/Professional Role and Identity**  *A coherent set of behaviors and displayed personal qualities of an individual in a social or work setting* | **Precision Oncology is Changing Pathology**  *It used to be where acute myeloid leukemia was classified according to the morphology or pattern of immunohistochemistry and now that is out of the window. Now it’s whether it has this or that translocation or this or that rearrangement.* [Non-rural Pathologist]  **Pathologists Envision a New Role**  *The time of [the] pathologist that hides in his or her office and gets the slides passed under the door and doesn’t talk to anybody is over. We are here to talk to you because it’s for the benefit of our patients and for our own edification and to support you*. [Non-rural Pathologist]  *We have between one or two tumor boards a day every day, and […] I attend all of them even though if I don’t have any case, often they ask me, “What do you think? Should we send this for profiling?” I’m available to them. Some rare tumor types are sometimes presented that I don’t know, and I say, “I don’t know. I will do research and get back with you*.” [Non-rural Pathologist]  *Ultimately, we are not only the stewards of the tissue but we’re also the owners of the classifications and it is not enough anymore to be good at recognizing things under a microscope. We do understand what is driving these diseases and the relevance and mechanisms that are altered or disorganized with each one of these translocations with the exception of maybe somebody doing clinicals and ethics, we are the physicians that are closest to what is happening and most pathologists have a research background and some kind of familiarity with molecular and genetics. We have to. So I think we’re going to be the driving force.*[Non-rural Pathologist]  **Some pathologists desire more active role in choosing tests**  *[Oncologists] don’t always consult with me as to whether the testing is going to happen or not. It’s something actually I’ve been interested in changing. At this hospital...they don’t even call the pathologist. They just call the laboratory people and say “we want this one sent off to” for example NeoGenomics or FoundationOne and since they put in the order, they cut me out of that decision making process.* [Non-rural Pathologist] | **Precision Oncology is Changing Expectations for General Oncology**  *I mean it’s very, very hard to be a general oncologist anymore. The field has exploded with each cancer almost a unique specialty of its own so increasingly we will see that many, at least in the academic centers there are doctors who only see one type of cancer because the field keeps changing* [Rural Oncologist]  *I think the other problem is we have so many new agents coming out that for instance, when one percent of people have it, there’s less familiarity with the drug and so I’m a general medical oncologist. I probably know some areas better than others but I treat technically anybody who walks in the door with any type of cancer.* [Non-rural Oncologist]  **Multiple providers responsible for surveillance**  *And then I also call them about a week after they’ve been on the medication as a follow-up just to make sure things are still going good, checking in. Sometimes they have a coinciding appointment with a provider to …check in on side effects to see if there’s any sort of things going on that to be addressed at that one week point. But sometimes it’s just the phone call with me and then they don’t see the provider for like a month after being on the medication and so yeah, that’s where I can kind of step in to be like “oh, you’re having bad nausea and not able to keep anything down. Alright. Let’s talk through this” or “you’re getting dizzy and you don’t know what to do about that.”* [Non-rural Pharmacist] |
| *No excerpts reflecting the optimism domain were identified | | |

**Supplemental Table 2. Capability Determinants of Testing and Treatment**

| **TDF Construct** | **Testing Themes** | **Treatment Themes** |
| --- | --- | --- |
| **Knowledge**  *Awareness of the existence of something* | **Some physicians are unaware of need for testing**  *When cost is removed, there are still other barriers.  It may be that for some patients cost is a barrier but I suspect that other things are going on.  I think again it’s one of the – I’ll just say anecdotally I think some part of it is physician education.* [Non-rural Surgeon]  **Rapidly expanding knowledge is challenging.**  *Unfortunately tumors are far more complex and you have many, many pathways and invasion of what we think is one driver kind of a pathway but yes, we still have for lung cancer, the care has been revolutionized. What started off with one or two targeted, now we have four or five different targets.* [Rural Physician]  *This field is rapidly advancing, and I say rapidly, would emphasize that daily... If I go on vacation and come back, I say, “What? What happened? What did I miss?”* [Non-rural Physician]  **Don’t know treatments for all markers**  *Obviously, we’re early in the field and there’s just a lot of mutations and some of them are important and some of them aren’t and we don’t know necessarily which ones are and which ones aren’t and so many of the mutations just don’t have a drug for it yet*. [Non-rural Oncologist] | **Rapidly expanding knowledge creates challenges, especially for community oncology**  *It’s definitely very challenging with so many new oncological drugs being proved every year, to keep up with most the up-to-date treatment…I’m also practicing in a community setting, so I treat all cancer types, so that means I have to keep up to date with all cancer types, not just like I treat just one or two*. [Rural Oncologist]  *I think the problem is in all honesty if you say can I know every single side effect of all of these drugs, every drug interaction and that sort of thing, the honest truth is I don’t know that I have a super solid working knowledge of all 50 drugs that were approved last year.*[Non-rural Pharmacist]  **Reports Presume Knowledge**  *I mean there are some nuances in that report that a person who is just trained in taking care of patients isn’t going to get and they’re also going to tell you that’s a mutation and there’s a drug for it but that’s not likely to be the driver so you’re not going to get a lot of bang for your buck by finding that drug and giving it to the patient. There are some actual interpretations that if you’re not getting some expertise in evaluating what they give you […]you’re going to make some mistakes because you’re not a molecular geneticist oncologist.* [Non-rural Oncologist]  **Knowledge begets new questions**  *when I talk to patients they’re like “well how long am I on this” especially those that have gone through chemotherapy in the past, “well how many months do I this” and it’s “no, I’m sorry, this isn’t a month long or a six month long process.  This is you’re going to be on this medication until you either can’t tolerate it or you have serious progression.”  So yeah, that can be a downside because people are like “oh, what do you mean I’m on this?  What do you mean you don’t know how long I’ll be on this for?* [Non-rural Pharmacist] |
| **Behavioral Regulation**  *Anything aimed at managing or changing objectively observed or measured actions* | **Offering tests is professional obligation**  *Really truly if you are familiar with the College of American Pathologists, some of the checklist for cancer reporting…require you to run most of these biomarkers. In other words, it is standard practice. You have to offer these things and you have to offer them in an organized manner because that’s the right thing to do*. [Non-rural Pathologist]  **Standard protocols would help, but they are difficult to develop**  *It comes up pretty frequently, but there’s still not a standard of care for what type of targeted panels are going to be ordered immediately, like without having to be specifically ordered.*[Non-rural Nurse]  *They get updated quite often so I think it’s the best way to ensure that my reports have all the information that is needed.* [Non-rural Pathologist]  *Now, we have so many more oncologists and pathologists spread throughout the state. I think it’s more difficult to come up with that, to overcome personal preferences, and come up with a standard protocol. There have been attempts to do it in the past, which have failed, because one or more people just were not on board, and it just fell through, so I would prefer that. I think it would deliver better care, so we’ll see.* [Non-rural Pathologist]  **Pathways changed practice**  *We’ve been on the ClinicalPath module since […] last year. […] There was some frustration on the physicians’ part initially […] it wasn’t easy, but now I think that the providers have kind of gotten used to it. They know what to expect. They know what the system is going to ask, and they just kind of go through it because it’s kind of like top of their mind now what they need to have ready to go when they’re going to make those treatment decisions.* [Non-rural Pharmacist]  **No information about how they are performing is coming back to them**  *Our data is growing thousands of patients, so we need someone to spend time to analyze that because if somebody analyzed those -- and I do that, but it’s just case by case because I’m the only one.* [Non-rural Pathologist] | **Pathways help facilitate guideline-concordant treatment and manage cost but may disrupt clinic flow without standardized testing**  *I think this new tool has kind of forced us to be more proactive than reactive on that front because they’re coming out every quarter with updates. Pathway is staying on top of these things to make sure that those new biomarkers are getting added to the pathway and guiding potential treatments for the patients* [Non-rural Pharmacist]  *That’s somewhat easier said than done as well because a lot of the times when you’re seeing that patient in consult, you don’t have all of that information and you’re trying to plug in pertinent information so that pathways come up and you don’t have this molecular test result back yet and just the flow of that. […] all of that could be – it needs to be smoother but it is what it is. I’m not sure how to make that work exactly perfectly. But that is a challenge.* [Non-rural Nurse]  **Online References and templates help**  *There’s a couple of websites that I kind of always have open at the ready, Up to Date, HemOnc.org are quick references that we use regularly here. Any of the NCCN provided template products that they supply are always really helpful for a quick and easy reference but I’d say the backbone of what I try to implement into practice comes from board certification for sure.* [Non-rural Pharmacist]  **NCCN Guidelines are Well Accepted**  *At least here, everybody definitely pays attention to the NCCN guidelines.  Granted, they are guidelines, and so they can certainly use their clinical judgment otherwise.* [Non-rural Nurse]  *So, I always almost have NCCN guidelines pulled up in my desktop here*. [Rural Oncologist] |
| **Memory, attention, decision processes**  *Ability to retain information; State of awareness in which senses are focused selectively on environment; extent to which a person can concentrate on relevant cues; cognitive process of choosing between two or more alternatives* | **Variability in test result formats and filing create difficulty acting on results**  *So we do a variety of next gen sequencing tests so … they’re coming from a variety of places and they show up, they seem to show up differently in EPIC.* [Non-rural Pharmacist]  *another huge challenge that I find is that because these results for the most part are specialty labs, they do not come into our Epic system in a discrete field and what a huge pain.  If that could be fixed across the world that would be great.* [Non-rural Nurse]  **Differentiating 1^st^, 2^nd^, 3^rd^ line treatments is difficult**  *I think part of that comes with practice and just knowing where care is moving for a certain disease state.  Breast cancer is looking for a HER2.  AML is looking for a FLT3.  Looking for KRAS in some of the colorectal cancers.  But when we start getting a little deeper into treatment, second opinions, third lines, fourth lines, no, I wouldn’t necessarily know.* [Non-rural Pharmacist] | **Precision oncology is beyond memory of any one individual**  *The volume of new information that’s coming out is always challenging to deal with and I think we’re kind of getting to an inflection point where it’s hard to have some of these things as memorized as you expect that you should.* [Non-rural Pharmacist]  **Behavior is too rare to keep in memory**  *…using those drugs is a big problem especially when any given provider and any given staff person is probably only going to see a couple of people getting those drugs over a couple of years*. [Non-rural Oncologist]  *it really depends on what’s on my radar. Again, our group doesn’t technically specialize, […] I think it’s just based on the referral patterns. Am I always on the cusp of the latest and greatest when breast changes? No. […]if it’s stuff where again you don’t necessarily know those guidelines like the back of your hand, […] Then it’s like okay, let’s check the guidelines and see what do they really say.* [Rural Nurse] |
| **Skills**  *Ability or proficiency acquired through practice* | **Skills navigating communication and relationships among different professional groups: pathologists, clinical genomics scientists, oncologists, etc.**  *We have a great working relationship with our pathology group. They’re very open to certainly as all of this is constantly changing, they change as well based on NCCN guidelines and recommendations as to what they reflexively test for so that we’re not – that’s been a big improvement as well that docs aren’t the ones having to know all this stuff and constantly be ready to order ALK and ROS and EGFR. Some things now, if it’s a lung cancer patient, it reflexively is being performed and sent out by pathology. That’s also helped speed up the process…So reflexive testing and a relationship with your Pathology Department I think is key so that you’re getting those things.* [Non-rural Nurse]  **Tumor board helps develop skills**  *I think the key is communication.  I think hearing what the oncologists are going through, also I think as much as I hate going to tumor board, it’s a lot of work, I think it is extremely helpful because they ask for the latest thing that they heard in one of the conferences and then we can look if that is reflected on our protocols or if not do we need to change it.* [Non-rural Pathologist]  **Skills communicating with patients about testing are necessary**  *I think they confuse it with genetic, like the normal genetic studies that we have been telling them, inherited cancer and all that. I try to make a very unique distinction about what does it really mean, and it takes time, but eventually they know we’re talking about genetics inside. I tell them like, “Inside your cancer cell, not that your mom or dad actually passed this to you but actually how your cancer [changes],” and then they tend to [understand].* [Rural Oncologist]  *I talk to them, “I’m going to request this test.  It’s going to tell us how to treat your cancer differently than chemotherapy.”  That’s exactly the word I use, “Different” like chemotherapy because I tell them, “If there is a chance that we will not treat you with chemotherapy, it’s because it will be with better outcomes and perhaps actually even better tolerability of your symptoms and all that.  That’s going to be very good for you.”  […].  In that way, I am walking through them to why, and I’m telling them why and how is it very, very important.* [Rural Physician] | **Skills navigating communication and relationships among different professional groups: pathologists, clinical genomics scientists, oncologists, etc.**  *And then in terms of kind of with providers, there have been some actually quite recently – in general they do rely on us quite a bit, us pharmacists to discuss “hey, this patient is having this type of reaction having these side effects, what do you think is going on?”  We’re talking with them pretty much on a daily basis whether face to face or they’re spending all of us messages through – we just transitioned to Microsoft Teams as our messaging system and so yeah, we’re very much talking with them.*  *It was tried for a while although I think it was really hard to keep up on it.  We were trying to send out emails of updates on new therapies coming out and new indications for older therapies already approved.  I think it was a little hard for the providers to keep up on all of those emails coming out but we do. A*dd w*ho said this*  **New skills communicating with patients about targeted treatment are necessary**  *this is the way I describe [targeted therapy] to a patient […]this is an analogy I use […] I tell them that number one is that I call chemotherapy or the regular treatment, I tell them that it’s more like a shotgun approach that is you’re blasting the cancer but unfortunately there’s a lot of collateral kind of damage. […] I mean with chemotherapy that’s what you’re creating with controlled poisoning.  […] And I tell them that targeted treatment is like a sniper bullet.* [Rural Physician]  *We do have pharmacists that have started being more a part of the education process and as part of our specialty pharmacy. […]  So, lots of different ways that we’re trying to be able to educate those patients on oral agents.* Non-rural Nurse] |

**Supplemental Table 3. Opportunity Determinants for Testing and Treatment**

| **TDF Construct** | **Testing Themes** | **Treatment Themes** |
| --- | --- | --- |
| **Environmental Context**  *Any circumstance of a person’s situation or environment that discourages or encourages the development of skills and abilities, independence, social competence, and adaptive behavior* | **Some community hospitals have less capacity for in-house testing**  *I think it’s the cost, if it’s cost effective, because some bigger institutions, they have a lot of patients that it makes sense to do it in-house. But for us and as a community hospital, I don’t think it’s feasible.* [Non-rural Molecular Biologist]  *It’s probably more expensive than it would be if all this testing was in-house but I guess that’s the unfortunate reality of being in a smaller practice.* [Non-rural Pathologist]  **Preauthorization is barrier to reflex testing**  *We have to test the tumor to see what kind of genetic markings it has on it…There is a lot of cost to this and the hospital will require a preauthorization from an insurance company before they will agree for us to send it to testing. … rather than reflexively sending a tumor once we have made the diagnosis to a laboratory to do the testing, we have to wait for preauthorization where some nonmedical person makes a determination as to whether it will be preauthorized or not.* [Non-rural Pathologist]  **Reimbursement and cost impede testing**  *Some insurance companies, they don’t pay at all, so we have that protocol, also.  We just don’t want a patient to end up with the bill, so we try to avoid those insurance companies.  We usually have a note in our pathology report that, “This was eligible for testing, but it was not sent.  Prior authorization required.”* [Non-rural Administrator]  *these tests are ten to twelve thousand dollars and sometimes it’s not covered by insurance so then […]it’s recommended by the guidelines but from a financial perspective maybe that’s not feasible.* [Non-rural Oncologist]  **Availability of clinical trials provides workaround to reimbursement limits**  *… it’s expensive. It typically runs about, again, the number changes but it’s like six or seven thousand dollars at least as it came out and again working at our hospital, I would have all kinds of people who didn’t have insurance and there’s no way they could afford seven thousand dollars for the test so we would usually put those people on the NCI-MATCH trial.* [Non-rural Oncologist]  **Delay in ordering tests because of Medicare 14-day rule**  *So because of Medicare rules that state that when the patient is still in the hospital their molecular testing cannot be sent until after 1*4 *days after discharge, many of the molecular tests … the Pathology Department became kind of a cost center for the hospital…What happens is that we can delay the tissue from being sent until the 14 days are done.* [Non-rural Pathologist]  *Obviously, the hospital doesn’t want to eat the cost for sequencing so we’ll wait 14 days and then send a sample.* [Non-rural Surgeon]  *It’s actually really counterproductive for patients because some of them are not in a stage in which they can wait two weeks for a result or actually it’s not two weeks.  It’s two weeks plus the week or ten days that it takes to run the results.* [Non-rural Pathologist] | **Insurance coverage for drugs is variable**  *Everybody’s insurance is very different on how they cover these medications but on average copays or just a month’s supply, so somewhere between 28 to 30 days of medication an average price I would say is probably one to two grand*. [Non-rural Pharmacist]  *With the assistance, I’ve had as little as zero copays to maybe $100 a month copay; whereas, without assistance, a lot of the patients will tell me, “I couldn’t do it because it’s like $3,000 for a month.”* [Rural Nurse]  **Only Certain Drugs are Reimbursed**  *I mean if you have something that is clearly a mutation that you ought to be looking for in a disease and you have a drug and it’s approved for that mutation and that disease, you don’t have a lot of problem. It’s only when you have a mutation and it’s approved and it’s approved for that mutation but it’s not approved for that mutation in that disease that you have issues.* [Non-rural Oncologist]  *...sometimes we might find the target but in the wrong cancer. These are very expensive so the insurance is obviously trying to find an excuse to cover it and I don’t blame them.* [Rural Oncologist]  **Rural areas/community hospitals may experience limited access to drugs and shoulder cost themselves**  *Some of the insurances have been more open to getting treatments in the rural areas. Some of them still require a lot of prior authorizations or clinical review to make sure it’s something that they really think the patient needs before they’re willing to reimburse us to giving it. It seems like if you’re in the bigger facility, they don’t question as much about the clinical review and the authorizations as much as they do in the rural settings.* [Rural Nurse]  *Well, specifically Opdivo, it’s not one that we can get here. Our wholesaler doesn’t carry it, so that goes through the city. Keytruda is one that we don’t get here for the same reason. It’s not available through our wholesaler, too. We’re a smaller hospital, so that’s kind of the issues with most of our targeted therapy.* [Rural Nurse]  **Community clinics may not have staff to optimize targeted therapy**  *There are certain differences […] between how we do it in the [hospital] clinic, in the city versus in the rural areas. […] the pharmacist in [hospital] here is more involved. They always contact the patient to make sure how they’re taking the drugs and any side effects so that’s a separate kind of involvement which I obviously can’t do all that in the rural setting and so there’s a lot more monitoring going on in the [hospital] setup.  […]  Then I also have a nurse practitioner who will commonly see them prior to starting them on treatment for doing a teach appointment.  We do that to tell them about all the side effects which is usually what I tell them when I see patients but this is a more comprehensive teach appointment and then they follow up with them a few weeks later to make sure they’re tolerating it.  There are several layers of protection which we cannot do in a rural setting.* [Rural Physician]  *Now we do have oncology pharmacists that help […] saying oh, by the way, did you know that you needed to change the dose of this because they are on this drug and all that sort of thing. I think we are fortunate that we are a pretty big center and we can afford dedicated oncology pharmacists. I think many places don’t have that luxury.* [Non-rural Physician]  **Providing targeted therapy in rural communities is important**  *I have a […] couple […]they’ve come 70 miles away to receive care at our center.  And it was because they didn’t want to drive the interstate.  And so they went to the [cancer center] in [city], which is world renowned in their abilities to care for patients there.  [But] those patients, that couple, they weren’t comfortable.  They weren’t comfortable driving to the care.  And if that’s going to be that feeling that they have every single day coming for treatment, and he was a combination of radiation and oncology, it’s not going to be a good outcome for them.  So, what they ended up doing was they referred them to us here in [city].  And even though it was a 70 minute drive, it was a drive they were comfortable doing.* [Rural Administrator] |
| **Resources** (a subset of Environmental Context and Resources) | **Tumor boards provide opportunities for learning and discussion, but molecular tumor boards and expertise are only at some sites.**  *We have a tumor board that lasts an hour once a month and we bring cases to that board to kind of go over that as well as to give us the actual mechanisms, the molecular mechanisms, all of the mutations and their downstream effects and what not* [Non-rural Oncologist]  *We do not have a precision medicine molecular tumor board* [Non-rural Pathologist]  *We have about 32 specialty tumor boards a month that I present at all of them. If a patient has molecular result, we present. I also have my own molecular tumor board, which is once a month.* [Non-rural Molecular Biologist]  **Testing companies provide tumor board staffing**  *They will help any group coordinate a molecular tumor board and be on the call and help review those results. At any time, certainly we could continue our molecular tumor board without them but it’s just been I think they’ve enjoyed it. It’s been great for us all to learn together and for their scientists sometimes to hear from the clinician perspective. That’s always good to have those physicians in day-to-day practice and then the physician scientists to meet in the middle sometimes about the challenges that are out there with the tests and interpreting the tests.* [Non-rural Nurse]  **Electronic databases and guides of genomic tests are helpful**  *Having the information electronically imported, not scanned. Large scale genomic testing in a unified database. The changes that are coming to like liquid biopsy I think are interesting where you can do large scale testing with one sample. You don’t always have to go back and get tissue or tissue isn’t the issue since you have a sample but really just having a single source for results to flow into.* [Non-rural Pharmacist]  *so just having, like I said, a centralized repository of that information because it takes time to gather. Our board recertification and certification info at best it’s updated once a year and that’s if you pay for that new information once a year but you don’t have to get recertified every year so a lot of times you don’t pay for it except every couple of years.* [Non-rural Pharmacist]  *I am kind of thankful for the ClinicalPath system. Honestly, it keeps me up to date* [Non-rural Pharmacist] | **Access to assistance programs to help with drug costs is variable and resource intensive**  *If they can’t afford it, the drug companies are actually fairly good about providing patient assistance. Some people are a little bit leery. To do that you’ve got to give them a lot of information and a lot of people don’t want to give their tax returns, etcetera but if they do, I find that cost is less of a barrier than I would have anticipated.* [Non-rural Oncologist]  *I would say it’s probably, I would say seventy-five percent of our patients we can usually like the grant route and yeah, we’ve got, yeah, the rest that are usually getting the medication through the manufacturer.* [Non-rural Pharmacist]  *Part of the role where specialty pharmacies come in […] is also being financial assistants and advocates. We work with patients to identify unaffordable copays[…] The unfortunate part is the grants from the foundations open and close depending on how much funding is available so they’re not always there. […] It’s ever changing. It could be there’s no funding available at nine am on a Monday and at two pm all of a sudden it could be open again and we have funding and then it’s just everybody is racing, all of these specialty pharmacies across the country, we’re constantly refreshing pages and we’re getting emails with updates at regular intervals and so yeah, it’s kind of been like a free for all of everybody rushing to go sign up.* [Non-rural Pharmacist]  ***Patients on oral therapy miss out on treatment monitoring which is organized through Infusion Clinics***  *I feel that one of the areas where we have a gap and a potential fall through in patient education is in oral agents because lots of times those folks don’t flow through the infusion area like our other patients do and, therefore, they miss that interaction with the chemotherapy infusion nurses who do a wonderful job at education..* [Non-rural Nurse]  *I think that the infused drugs just inherently come with more support.  You develop more of a rapport with the patients because they’re here more often, and you can assess them more for subtle changes […]If there are side effects, you know what kind of adjustments to make.  If somebody sits home with pills, you’re not going to see them for 30 days perhaps.  Even though you tell them to call with any problems, and you give them specifics, they rarely don’t, so they end up in the hospital, which is never what we want.*[Non-rural Nurse]  **Need simple “cheat sheet”**  *if there was a list of common disease states, lung, breast, colorectal, melanoma, unless it tends to have driver mutations, the list and then the most common mutations, the implications of those mutations, whether it’s a poorer outcome and some sort of percentage, likely treatments that are associated with those particular mutations. […], but I would say just like some small, one sheet of paper organized, just a cheat sheet almost that’s for somebody that’s not at doctorate level.* [Non-rural Nurse] |
| **Social Influences**  *Those interpersonal processes that can cause individuals to change their thoughts, feelings, or behaviours* | **Communication and relationships among different professional groups: pathologists, clinical genomics scientists, oncologists, etc.**  *we try to have it less technical because the physicians are super smart, but they don’t have training in the field that I was trained for […] I think that the collaboration between their knowledge about treatment and my knowledge about the science and the background, I think that helped both of us to learn more*. [Non-rural Molecular Biologist]  *The oncologists I work with now have a lot of different personal preferences…so that that’s why I ask. Anything besides the breast cancer standard biomarkers or MMR in endometrial cancers and colon cancers, I always have to ask.* [Non-rural Pathologist] | **Communication and relationships among different professional groups: pathologists, clinical genomics scientists, oncologists, etc.**  *A lot of times depending on the importance of the drug, there’s a number of anti-epileptics that have a lot of potential metabolism changes with certain oral targeted therapies.  I can always start the conversation but an oncologist isn’t always necessarily willing to mess with somebody’s anti-epileptics without ...of another neurologist or other neurologist but what’s nice is I can facilitate that conversation a lot of times or I can act on behalf of the oncologist to start that conversation.* [Non-rural Pharmacist]  **Patient influences treatment in both directions**  *I feel like most of the patients strongly prefer the oral tablets.* [Rural Physician]  *if I’ve got people that I don’t trust to take the pills, I’m not going to give them the pill option.* [Rural Nurse]  **Knowledge is gleaned from multiple trusted sources**  *At our office and this is different and I think this is probably office to office but this is different than when I was in [city], when I was in [city] at least the group with whom I worked at [hospital] and that was 10 to 15 years ago so but they would let reps into the office. Here there are no reps in the office. None at all. All of the reps have to go through our pharmacist and if they want to provide information to the group that’s how that is done. That gets filtered through our pharmacist. She’ll say “hey, I met with John Smith with ABC Drug”. There’s some of that. So we get some of that. Certainly some of it comes through journals that I get at home. Some of it is email that I get at home for different organizations with which I belong. Some of it is just periodically honestly checking the NCCN guidelines and have they changed because sometimes I feel like they change so quickly.* [Rural Nurse] |
